# Screening and Evaluation of Salt-Tolerant Wheat Cultivars

**DOI:** 10.3390/plants15121873

**Published:** 2026-06-17

**Authors:** Hongping Chang, Wei Fu, Zhichao Zhang, Shucan Liu, Wenjun Xiao, Ruize Qin, Qinghua Lu, Xinhong Guo

**Affiliations:** 1 College of Life Science and Engineering, Handan University, Handan 056005, China; hanghongping123@hdc.edu.cn (H.C.); hdxyfw@126.com (W.F.); zhangzhichao202604@126.com (Z.Z.); 2 Institute of Bast Fiber Crops, Chinese Academy of Agricultural Sciences, Changsha 410205, China; liushucan@caas.cn; 3 National Center of Technology Innovation for Saline-Alkali Tolerant Rice, Sanya 572024, China; wenjun_xiao@126.com; 4 College of Biology, Hunan University, Changsha 410028, China; miaomiao@hnu.edu.cn

**Keywords:** wheat, salinity tolerance, antioxidant enzymes, physiological and biochemical indicators, cultivar screening

## Abstract

Wheat, a global staple crop, faces severe yield threats from abiotic stresses like salinity. In this study, salt tolerance was assessed in five wheat cultivars from the Huang–Huai–Hai region of China (*Ji 6358*, *Jimai 22*, *Keyi 5214*, *Liangxing 99*, and *Nongda 3432*) by exposing seedlings to NaCl concentrations ranging from 0 to 150 mM. Under 150 mM NaCl treatment, *Liangxing* 99 and *Jimai* 22 displayed greater root and shoot growth, accompanied by more biomass accumulation than *Ji* 6358. Physiological and biochemical indicator assays revealed that under 100 mM and 150 mM NaCl treatment, *Liangxing 99* and *Jimai 22* exhibited significantly higher levels of chlorophyll, proline, and soluble sugars, coupled with lower MDA accumulation than *Ji* 6358. Additionally, the activities of antioxidant enzymes, including SOD and CAT, were highest in *Liangxing 99* and *Jimai 22*, consistent with the lowest H_2_O_2_ accumulation observed among the five wheat cultivars under 150 mM NaCl treatment. qRT-PCR analysis of *Liangxing 99* and *Jimai 22* under 150 mM NaCl treatment revealed that transcript levels of the abiotic stress-responsive gene *ZFP34*, antioxidant-related genes (*APX1*, *FSD2*, *APX2*), and the photosynthetic gene *RbcS* were significantly higher in *Liangxing 99*. These findings demonstrated that *Liangxing 99* exhibited relatively strong salinity tolerance among the five tested wheat cultivars. This study offers insight into the identification of elite salt-tolerant wheat cultivars at the seedling stage and provides valuable guidance for future breeding programs aimed at enhancing wheat productivity in saline environments.

## 1. Introduction

Hebei Province is located in North China and belongs to the Huang–Huai–Hai wheat production region, which includes Hebei, Shandong, Beijing, and Tianjin. *Liangxing 99*, *Jimai 22*, *Nongda 3432*, *Ji 6358*, and *Keyi 5214* wheat cultivars are suitable for planting in the Huang–Huai–Hai region. They differ in genetic background, growth duration, plant height, and yield traits, and their salt tolerance has not been systematically evaluated and compared. To screen wheat cultivars adaptable to saline lands in the Huang–Huai–Hai region, these five cultivars were selected as experimental materials. We hope our findings can deliver practical guidance for local cultivar application and salt-tolerant wheat breeding.

Wheat is a worldwide staple food, but its production is significantly threatened by abiotic stresses such as drought, salinity, and high temperature, which pose serious risks to global food security [1,2,3,4,5,6]. Under adverse stresses, plants can perceive and transmit stress signals, activate response transcription factors, activate the transcription of stress-responsive genes, and trigger a series of physiological and biochemical reactions to resist the harm of adverse environments [7]. Currently, about 7% of the global land area is affected by soil salinity, covering an area of 950 million hectares [8,9]. Projections indicate that global climate change will increase the frequency of extreme weather events such as drought and high temperature [10,11]. These two stressors will further drive the continuous expansion of global irrigation systems, thereby exacerbating land salinization. Currently, 52% of the global population across 13 countries is suffering from severe land salinization [12]. With the gradual depletion of freshwater resources, modern agricultural production has become increasingly vulnerable to salt stress [13]. Accordingly, enhancing salt tolerance in wheat is becoming increasingly critical.

Salinity is one of the major abiotic stresses that significantly affect crop productivity worldwide [14,15]. The adaptive response to salt stress can be divided into three processes: osmotic stress regulation, ion homeostasis maintenance, and reactive oxygen species (ROS) detoxification [9]. Salt stress adversely affects seed germination, plant growth, and reproductive development and can even cause plant death under severe stress conditions [9]. Salt stress impairs the water uptake capacity of plant roots. Meanwhile, root-absorbed salts are transported to leaves via the transpiration stream; excessive cytoplasmic Na^+^ suppresses the absorption of essential ions and disrupts core metabolic pathways [9]. Under salt stress, plants also accumulate ROS, leading to oxidation of cell membranes, proteins, and DNA, ultimately resulting in cell death [16]. To adapt to salt stress, plants have evolved sophisticated tolerance mechanisms involving physiological, biochemical, and morphological modifications [17].

To alleviate osmotic disturbance under salt stress, plants accumulate a wide range of organic osmolytes that protect cellular structures and sustain regular metabolic activities. Proline is one of the most important osmolytes for osmotic adjustment in plant cells [18]. As a multifunctional osmotic regulator and cellular protectant, it preserves osmotic balance, reduces membrane injury, and eliminates excess ROS [19]. Under normal conditions, the level of free proline in plants is relatively low. However, under adverse conditions such as drought, low temperature, and high salt, proline accumulates extensively in plants [20]. The accumulation of proline is a sign of plant tolerance to abiotic stress through different stress signaling pathways (such as plant hormones, calcium signaling, and mitogen-activated proteins) and is positively correlated with stress resistance. Therefore, proline is used as one of the biochemical indicators of plant stress resistance [20,21].

Salt stress disrupts osmotic balance and elicits oxidative injury in plants. Malondialdehyde (MDA) serves as a key biomarker to quantify oxidative stress, and its content rises markedly under salinity due to intensified oxidation [22]. As a major abiotic stress, salt stress restricts plant growth and causes oxidative damage mainly via excessive ROS [20,21,22]. These oxygen-derived reactive molecules include O_2_^−^, H_2_O_2_, and·OH. Accumulated ROS damage proteins, lipids, and nucleic acids, disrupt cell metabolism and homeostasis, and initiate aberrant cell death [23]. Plants rely on enzymatic and non-enzymatic antioxidant systems to resist oxidative damage via improved ROS scavenging and modulated expression of stress-responsive genes [23]. As core components of this defense network, antioxidant enzymes, including SOD, CAT, and POD, act to remove excess ROS and alleviate oxidative stress [24].

Previous studies on wheat salt tolerance have mainly focused on large-scale germplasm screening, single-stress physiological analysis, or the molecular mechanism of a single wheat genotype. Most published studies have broadly explored wheat salt responses without specifically targeting the commercial cultivars currently cultivated in the Huang–Huai–Hai region. To fill these research gaps, the present study evaluated the seedling-stage salt tolerance of five widely cultivated wheat cultivars (*Ji 6358*, *Jimai 22*, *Keyi 5214*, *Liangxing 99*, and *Nongda 3432*) in the Huang–Huai–Hai region under gradient NaCl treatments (0, 100, and 150 mM). This work has unique practical value for assessing these local dominant cultivars and informing wheat production in saline areas of the Huang–Huai–Hai region.

## 2. Results

### 2.1. Phenotypic Analysis of Different Wheat Cultivars Under Salt Stress

**Figure 1:**
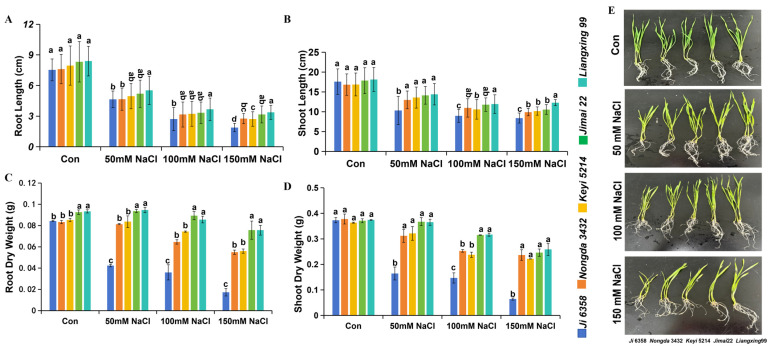
Phenotype of different wheat cultivars under salt stress. (**A**–**D**) Root length, shoot length, root dry weight, and shoot dry weight measured at 14 days after different salt stress. (**E**): Phenotype of different wheat cultivars under salt stress. Seedlings of different wheat cultivars were grown for 2 days on NaCl-free Hoagland solution and then transferred to Hoagland solution with or without NaCl. Values are means ± SD of 60 seedlings. All significant differences among different wheat materials under normal control and salt treatment conditions were determined by one-way analysis of variance (ANOVA), and Tukey’s multiple range test was further performed for pairwise comparisons at a significance level of *p* < 0.05. Different lowercase letters above the columns within the same indicator indicate statistically significant differences at *p* < 0.05 level according to Tukey’s multiple range test; values sharing identical lowercase letters show no significant difference between treatments.

Five wheat cultivars were treated under different salt stress (0, 50, 100, 150 mM); root length, shoot length, root dry weight, and shoot dry weight were analyzed, and the results are shown in Figure 1. Salinity significantly inhibited root elongation in all tested cultivars. Compared with the control group, the root lengths of *Ji* 6358, *Nongda* 3432, *Keyi* 5214, *Jimai* 22, and *Liangxing* 99 were reduced by 63.89%, 58.47%, 59.50%, 59.90%, and 56.27% under 100 mM NaCl, and by 74.95%, 63.80%, 65.83%, 61.93%, and 59.87% under 150 mM NaCl (Figure 1A). Shoot elongation of all tested wheat cultivars was obviously suppressed under NaCl stress. Under 150 mM NaCl treatment, the shoot lengths of *Ji* 6358, *Nongda* 3432, *Keyi* 5214, *Jimai* 22, and *Liangxing* 99 were reduced by 52.20%, 41.11%, 39.70%, 40.78%, and 32.11% (Figure 1B). In general, *Liangxing* 99 consistently possessed long roots and shoots, along with low growth reduction rates at 100 and 150 mM NaCl conditions. By contrast, *Ji* 6358 displayed serious growth inhibition. These results indicate that *Liangxing* 99 had relatively strong seedling salt tolerance among the five cultivars.

We further evaluated seedling biomass to quantify salt-induced growth limitation. Elevated NaCl concentration led to a gradual reduction in whole-plant biomass for all cultivars. At 100 mM NaCl, the extent of root dry weight reduction differed greatly among cultivars: *Jimai* 22 had the slightest decrease (3.60%), whereas *Ji* 6358 suffered a biomass loss of 57.31% (Figure 1C). For shoot dry weight, the reduction rates were 15.09% for *Jimai* 22 and 15.33% for *Liangxing* 99, lower than that of *Ji* 6358 (60.63%) (Figure 1D). Under 150 mM NaCl, the inhibitory effect was further intensified. *Ji* 6358 lost nearly 80% of root dry weight and over 82% of shoot dry weight. By comparison, *Liangxing* 99 only presented 18.93% and 30.84% reductions in root and shoot dry weight, respectively (Figure 1C,D). Collectively, biomass characteristics differentiated salt tolerance levels of the five cultivars and indicated that *Liangxing* 99 and *Jimai* 22 possessed stronger salt tolerance than other cultivars.

### 2.2. Physiological and Biochemical Indicators Analysis of Different Wheat Cultivars Under Salt Stress

**Figure 2:**
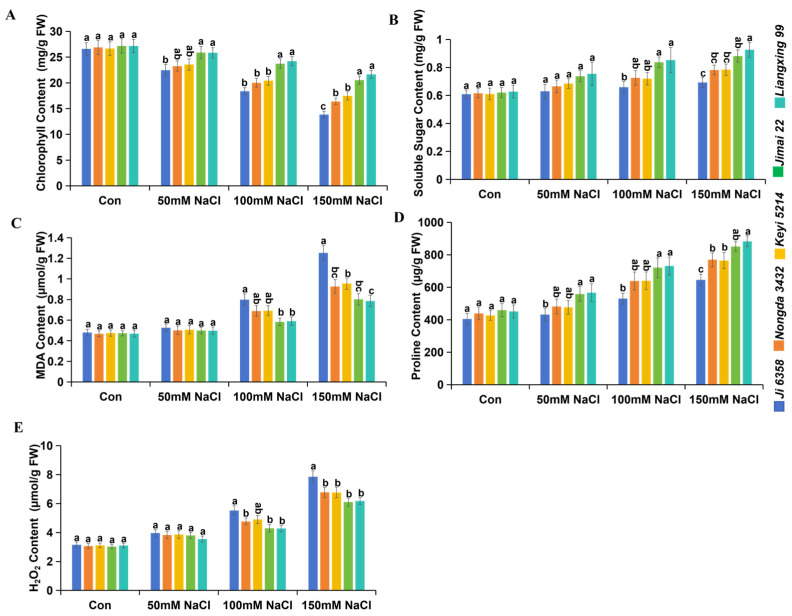
Physiological and biochemical indicators of different wheat cultivars under salt stress. (**A**–**E**) Chlorophyll content, soluble sugar content, MDA content, proline content, and H_2_O_2_ content measured at 3 days after different salt stress. Whole wheat seedlings were used for the determination of soluble sugar content, MDA content, proline content, and H_2_O_2_ content, while leaves were used to measure chlorophyll content. Values are means ± SD of three repeats. All significant differences among different wheat materials under normal control and salt treatment conditions were determined by one-way analysis of variance (ANOVA), and Tukey’s multiple range test was further performed for pairwise comparisons at a significance level of *p* < 0.05. Different lowercase letters above the columns within the same indicator indicate statistically significant differences at *p* < 0.05 level according to Tukey’s multiple range test; values sharing identical lowercase letters show no significant difference between treatments.

Five wheat cultivars were subjected to salt stress at concentrations of 0, 50, 100, and 150 mM for 3 days; chlorophyll, soluble sugar, MDA, proline, and H_2_O_2_ contents were determined, and the results are shown in Figure 2. Under control conditions, the five cultivars maintained relatively stable levels of these physiological substances. After exposure to 100 mM and 150 mM NaCl, chlorophyll content decreased in all seedlings, and the magnitude of the reduction increased with rising salt concentration. *Ji* 6358 suffered the most severe chlorophyll loss, with a maximum reduction of 47.92% at 150 mM NaCl. By comparison, *Liangxing* 99 and *Jimai* 22 retained higher chlorophyll levels, with reduction rates of only 20.32% and 24.46% at 150 mM NaCl, indicating better preservation of photosynthetic pigments under salt stress (Figure 2A).

Salinity induced obvious accumulation of soluble sugar and proline, two key osmolytes for stress adaptation. *Liangxing* 99 recorded the highest accumulation of soluble sugar and proline at NaCl conditions, with respective increases of 35.69% and 61.99% under 100 mM NaCl, and 47.58% and 95.50% under 150 mM NaCl (Figure 2B,D). Strong osmolyte accumulation enabled the cultivar to effectively mitigate osmotic stress.

As indicators of oxidative damage, MDA and H_2_O_2_ contents rose sharply under saline conditions. *Ji* 6358 showed large increases in MDA and H_2_O_2_, reaching 161.86% and 149.63% higher than the control at 150 mM NaCl, which reflected severe oxidative injury. In contrast, *Liangxing* 99 and *Jimai* 22 showed the slightest increases in MDA and H_2_O_2_, indicating efficient scavenging of ROS and well-preserved cell membrane integrity (Figure 2C,E).

Overall, the physiological responses were consistent with growth performance. *Liangxing* 99 and *Jimai* 22 possessed superior photosynthetic stability, stronger osmotic regulation capacity, and better oxidative stress tolerance, which collectively underpinned their seedling salt tolerance.

**Figure 3:**
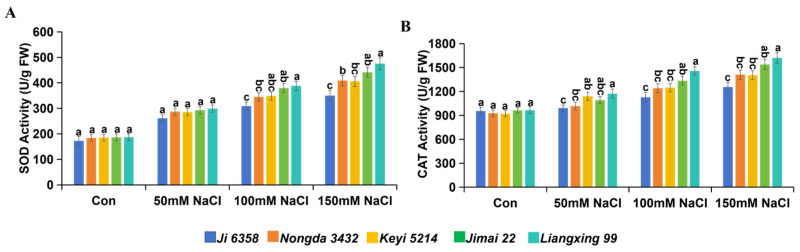
SOD and CAT activities of different wheat cultivars under salt stress. (**A**,**B**) SOD activity and CAT activity measured at 3 days after different salt stress. Whole wheat seedlings were used for this analysis. Values are means ± SD of three repeats. All significant differences among different wheat materials under normal control and salt treatment conditions were determined by one-way analysis of variance (ANOVA), and Tukey’s multiple range test was further performed for pairwise comparisons at a significance level of *p* < 0.05. Different lowercase letters above the columns within the same indicator indicate statistically significant differences at *p* < 0.05 level according to Tukey’s multiple range test; values sharing identical lowercase letters show no significant difference between treatments.

We further determined the activities of SOD and CAT, two antioxidant enzymes that constitute the major defense system against oxidative stress. Under non-saline conditions, SOD and CAT activities remained at relatively low and stable levels across all five wheat cultivars. After NaCl application, both enzyme activities were induced and rose continuously with an increase in salt concentration. At 100 mM NaCl, SOD activity increased by 78.65–107.51% relative to the control, while CAT activity increased by 17.97–50.76%. When salt concentration was elevated to 150 mM, the induction effect became more prominent: the rising range of SOD activity expanded to 103.49–154.05%, and CAT activity increased by 31.41–67.62% (Figure 3A,B).

Among all tested cultivars, *Liangxing* 99 exhibited the highest SOD and CAT activities at 100 and 150 mM NaCl conditions, accompanied by the largest relative increments in the five tested cultivars. *Jimai* 22 also maintained strong enzyme activities and considerable growth rates. In contrast, *Ji* 6358 showed the weakest enzyme induction capacity, with the lowest activity and the smallest increase amplitude in the five tested cultivars (Figure 3A,B).

The distinct differences in antioxidant enzyme activities well explained the varying degrees of ROS accumulation and oxidative damage among cultivars. Stronger activation of SOD and CAT enabled *Liangxing* 99 and *Jimai* 22 to efficiently eliminate excess ROS, which is an enzymatic mechanism supporting their superior salt tolerance.

### 2.3. qRT-PCR Analysis of Jimai 22 and Liangxing 99 Wheat Cultivars Under Salt Stress

**Figure 4:**
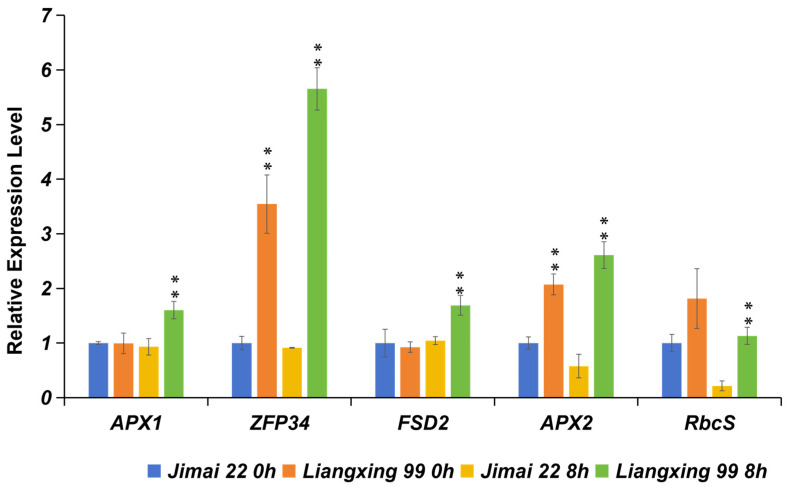
qRT-PCR analysis of stress-responsive genes in *Jimai 22* and *Liangxing 99* wheat cultivars under salt stress. Expression level of stress-responsive genes in *Jimai 22* and *Liangxing 99* wheat cultivars before and after 150 mM NaCl treatment for 8 h. Whole wheat seedlings were used for qRT-PCR analysis. Three biological replicates and four technical replicates were used in this analysis. Values are means ± SD of these repeats. Asterisks indicate significant differences between *Jimai 22* and *Liangxing 99* wheat cultivars (** *p* < 0.01, using Student’s *t*-test). *TaRP15* was selected as a house-keeping gene for qRT-PCR analysis.

Combined with seedling growth performance, osmotic adjustment status, antioxidant enzyme activity, and oxidative damage levels, our phenotypic and physiological-biochemical analyses collectively demonstrated that *Jimai* 22 and *Liangxing* 99 exhibited stronger salt adaptability compared with the other three wheat cultivars. To further explore the molecular regulatory mechanisms underlying their superior salt tolerance, these two salt-tolerant cultivars were selected for subsequent gene expression analysis. qRT-PCR analysis was performed on *Jimai* 22 and *Liangxing* 99 at 0 h and 8 h after 150 mM NaCl treatment. The results indicated that the expression levels of *APX1*, *FSD2*, and *RbcS* were significantly higher in *Liangxing* 99 than in *Jimai* 22 at 8 h after NaCl treatment. Additionally, the expression levels of *ZFP34* and *APX2* were consistently higher in *Liangxing 99* than in *Jimai 22* at both 0 h and 8 h of NaCl treatment (Figure 4).

## 3. Discussion

Salt tolerance in wheat is a quantitative genetic trait controlled by multiple genes, involving the coordinated action of multiple genes and salt tolerance mechanisms. Salt tolerance varies distinctly among different cultivars [25,26]. This study investigated the salt tolerance of five wheat cultivars (*Ji 6358*, *Jimai 22*, *Keyi 5214*, *Liangxing 99*, and *Nongda 3432*). After comprehensive analyses of phenotypic traits, physiological and biochemical indicators, and qRT-PCR assessments, our results indicated that *Liangxing* 99 exhibits relatively strong seedling salt tolerance among the five cultivars.

Salt stress inhibited plant growth. Comparisons of root length, shoot length, and biomass before and after salt treatment showed *Liangxing* 99 retained better growth performance, reflecting its superior salt tolerance. The slightly higher shoot length of *Liangxing* 99 seedlings was observed under 150 mM NaCl compared with the 100 mM treatment. It is supposed that minor random variations originating from seed vigor heterogeneity, microenvironmental inconsistency, and manual measurement contributed to this marginal difference, while such small fluctuations failed to reach a statistically significant level.

Chlorophyll is the material basis for photosynthesis in plants, and its content directly affects plant growth [27]. Previous studies have demonstrated that salt-tolerant wheat cultivars generally exhibit a slight reduction in chlorophyll content under salt stress [8,28,29,30]. Our results showed that leaf chlorophyll contents of *Jimai* 22 and *Liangxing* 99 were significantly higher than those of the other cultivars under salt stress, whereas their chlorophyll reduction rates were also lower compared with the remaining cultivars. This indicates that the two cultivars may maintain a higher photosynthetic rate under salt stress, suggesting they possess greater potential for salt tolerance.

When plants are exposed to unfavorable environmental conditions, they synthesize various osmoregulatory substances, including proline, soluble sugars, and soluble proteins, to adapt to stress. Proline exhibits potent osmotic regulation and strong antioxidant capacity, rendering it a vital physiological index to evaluate plant stress tolerance [31]. Salt stress can lead to excessive accumulation of ROS, disrupt ROS homeostasis, decrease lipid membrane function, and ultimately cause oxidative damage to plant cells. The concentration of MDA is an important physiological indicator of lipid peroxidation and changes in plant cell membranes under stress [32]. Salt can increase cell membrane permeability, enhance lipid peroxidation, and ultimately lead to membrane system damage. CAT and SOD are important enzymes in the biological defense system established during the process of biological evolution. It has been reported that salt-tolerant wheat cultivars accumulate higher proline levels, maintain lower MDA and H_2_O_2_ contents, and exhibit higher SOD and CAT activities under salt stress conditions [28,33,34]. In this research, *Liangxing* 99 and *Jimai* 22 showed relatively better osmotic adjustment, as reflected by their significantly increased contents of proline and soluble sugars under salt stress. These compatible osmolytes effectively lowered cellular osmotic potential, alleviated osmotic stress caused by salinity, and prevented severe water loss in seedling tissues. Meanwhile, they maintained relatively high chlorophyll levels, which suggested the normal progress of photosynthesis. By contrast, the remarkably lower MDA content in *Liangxing* 99 and *Jimai* 22 indicated that their cell membrane structures suffered less oxidative damage compared with other cultivars, which provided a physiological basis for sustaining normal metabolism under saline conditions.

Apart from osmotic regulation, the two cultivars also possessed a stronger antioxidant defense system. Salt stress induces excessive H_2_O_2_ production, which causes oxidative damage to plant cells. Here, *Liangxing* 99 and *Jimai* 22 maintained significantly higher SOD and CAT activities, which efficiently eliminated intracellular H_2_O_2_ and kept its content at the lowest level among all tested cultivars after NaCl treatment. Efficient H_2_O_2_ scavenging relieved oxidative stress and protected cell integrity. Collectively, coordinated osmotic adjustment and strong ROS detoxification jointly constitute the physiological mechanisms for the salt tolerance of *Liangxing* 99 and *Jimai* 22.

Ascorbic acid peroxidase (APX) is an important enzyme that can clear accumulated H_2_O_2_ in plants [35]. In *Arabidopsis thaliana*, there are eight members of the *APX* gene family, including *APX2* and *APX1* genes. AtAPX1 clears excessive ROS, preventing plants from oxidative damage [36]. Many *AgAPX* genes contain multiple cis-elements related to stress, and APX activity increases under high-temperature stress [37]. As a member of the iron superoxide dismutase gene family, FSD2 is predominantly localized in chloroplasts; its upregulation specifically targets the elimination of superoxide anion accumulated during photosynthesis under salt stress, forming a synergistic ROS-scavenging network together with APXs. CsFSD2 may participate in the clearance of ROS generated during various stresses and photosynthetic electron transfer under light, thereby protecting plants from stress [38]. The elevated expression of these genes in *Liangxing* 99 effectively alleviates oxidative damage. Consistent with the elevated activities of corresponding antioxidant enzymes, the increased transcript levels of these three genes enhanced the capacity to scavenge superoxide anion and H_2_O_2_. The effective removal of ROS prevents the peroxidation of cell membranes and protects the integrity of cellular structure, which in turn provides a foundation for the normal synthesis and accumulation of osmoprotectants. A stable osmotic environment further supports cell turgor and basic metabolic activities under salt stress. By contrast, the relatively low expression of these genes in *Jimai* 22 may lead to insufficient ROS elimination, disrupted cellular metabolism, and impaired osmotic regulation capacity.

*ZFP34* is a member of the C_2_H_2_ Zinc finger family. *ZFP34* was upregulated by dehydration, salt, cold, and oxidative stresses in wheat roots [39]. TaZFP34 upregulates the expression of the proline synthesis-related gene *P5CS*, which improves osmotic adjustment and reduces oxidative damage in wheat under salt stress [39]. As a regulatory factor, *ZFP34* does not directly participate in ROS detoxification, but modulates the expression of a series of downstream stress-related genes. ZFP34 may participate in the regulation of salt stress signal transduction, helping plants perceive and respond rapidly to a saline environment. The low expression level of ZFP34 in *Jimai* 22 may decrease this regulatory cascade, weakening the overall stress resistance of plants.

There are 20 *RbcS* (ribosomal binding protein S) family genes in the genome of *Triticum aestivum*. Members of this family play an important role in the adaptation to abiotic stress [40]. *RbcS* encodes the small subunit of Rubisco, an enzyme in plant photosynthetic carbon fixation. Its expression level is correlated with chlorophyll content and photosynthetic capacity. Under salt stress, the higher expression of *RbcS* in *Liangxing* 99 can maintain normal Rubisco synthesis and activity, which ensures efficient photosynthetic carbon assimilation. On the one hand, the antioxidant system regulated by *APX1*/*APX2*/*FSD2* and *ZFP34* protected chloroplast structure and chlorophyll from ROS destruction, creating a favorable microenvironment for the expression and function of *RbcS*. On the other hand, sustained photosynthesis provides sufficient energy and carbon skeletons for the synthesis of osmotic substances, forming a positive feedback loop between photosynthetic metabolism and stress adaptation.

This study revealed the physiological and molecular response characteristics of five wheat cultivars widely planted in the Huang–Huai–Hai region under salt stress. The results enrich the current understanding of salt tolerance mechanisms for local mainstream wheat cultivars. Furthermore, we analyzed the salt-tolerant performance of *Liangxing* 99. It exhibits strong ROS-scavenging capacity, stable photosynthetic status, and upregulated expression of stress-related genes under salt treatment, which demonstrates its potential as a parent material for salt-tolerant wheat breeding in this region.

*Liangxing 99* is a semi-winter wheat cultivar developed by Shandong Liangxing Seed Industry Co., Ltd. It has strong cold resistance, strong tillering ability, high panicle formation rate, excellent quality, and good yield performance. It has outstanding performance in Shandong Province and the Huang–Huai–Hai region. The Huang–Huai–Hai region is one of the main wheat-producing areas in China. *Liangxing 99* is one of the important parents for wheat hybrid breeding at present. Our results showed that the *Liangxing 99* cultivar had strong salt tolerance. This study provides crucial insights into the optimal utilization of *Liangxing 99* in future wheat breeding programs in the Huang–Huai–Hai region.

This study adopted merely five wheat cultivars from the Huang–Huai–Hai region, which fail to fully represent the genetic diversity of local wheat germplasms. The limited sample size may lower the generality and extrapolation reliability of the results.

All physiological and molecular analyses were carried out at the seedling stage only, so the data cannot reflect salt adaptability throughout the whole growth period. In addition, we did not detect ion contents. Since ion imbalance is a typical damage caused by salt stress, such data would help further elucidate the salt tolerance mechanisms of the tested cultivars. Our molecular research is also restricted to the expression analysis of five stress-related genes; further work including omics analysis and gene function verification is required. Additionally, all assays were completed under laboratory conditions, lacking validation in natural saline fields, where environmental factors are more complicated. Field experiments are therefore necessary to confirm the practical salt tolerance of these wheat cultivars.

In follow-up studies, we will expand the number of tested germplasms, perform multi-stage identification, detect ion dynamics, conduct in-depth molecular research, and carry out field trials to improve the current results.

## 4. Materials and Methods

### 4.1. Culture of Experimental Materials

Wheat cultivars *Ji 6358* (Hebei Woyu Agricultural Technology Co., Ltd. Shijiazhuang, China), *Jimai 22* (Hebei Letu Seed Co., Ltd. Shijiazhuang, China), *Keyi 5214* (Hebei Defeng Seed Co., Ltd. Shijiazhuang, China), *Liangxing 99* (Shandong Liangxing Seed Co., Ltd. Dezhou, China), and *Nongda 3432* (Hebei Defeng Seed Co., Ltd. Shijiazhuang, China) were selected as experimental materials. Seeds of these cultivars were washed three times, then placed at 4 °C for 3 days. Then, the seeds were put on wet towels for germination. Seedlings were cultured until the two-leaf and one-heart stage for further experiments.

### 4.2. Experimental Methods

#### 4.2.1. Salt Stress

Seedlings were subjected to Hoagland nutrient solution (Phygene Biotechnology Co., Ltd., Fuzhou, China) containing varying concentrations of NaCl (0, 50, 100, 150 mM), with a NaCl-free Hoagland solution serving as the control group.

#### 4.2.2. Statistics of Biomass

After salt stress, wheat seedlings were washed clean to measure root length, shoot length, root dry weight, and shoot dry weight.

#### 4.2.3. Chlorophyll Content Determination [41]

The collected samples were quickly frozen in liquid nitrogen, placed in a mortar, and ground quickly into powder. A total of 0.1 g of powder was weighed and added to 10 mL of80% acetone, placed on a shaker in the dark and shaken until the sample lost its green color, then measured at 663 nm and 645 nm.

Chla content (mg/g FW) = (12.7 A663 − 2.69 A645) × V × N / W  Chlb content (mg/g FW) = (22.9 A645 − 4.68 A663) × V × N / W  Chl content (mg/g FW) = Chla content + Chlb content

#### 4.2.4. Soluble Sugar Content Determination

Soluble sugar content was determined by the anthrone colorimetric method [42]. The plant sample was heated with concentrated sulfuric acid and anthrone, and then the absorbance of the generated purple product was measured by the colorimetric method. According to the standard curve, the soluble sugar content in the sample can be calculated.

#### 4.2.5. MDA Content Determination

MDA content was determined via the thiobarbituric acid (TBA) colorimetric method [42]. In total, 1 mL of plant tissue phosphate-buffered extraction supernatant was mixed with 2 mL of 0.6% TBA solution, then transferred to a centrifuge tube (sealed and heated in boiling water for 15 min) and quickly cooled and centrifuged. The supernatant was collected and determined at 600, 532, and 450 nm.

MDA content (µmol/g FW) = (6.45 × (A532 − A600) − 0.56 × A450) × V/W

#### 4.2.6. Proline Content Determination

Proline content was determined by the acidic ninhydrin colorimetric method [43]. The plant sample was heated with sulfosalicylic acid, and the extraction solution was mixed with glacial acetic acid and acidic ninhydrin. The mixture was then incubated in boiling water for 30 min until a red color developed, then cooled to room temperature, after which toluene was added. The mixture was shaken vigorously to extract chromogen, allowed to stand for stratification, and then the absorbance was measured by the colorimetric method. According to the standard curve, the proline content in the sample can be calculated.

#### 4.2.7. H_2_O_2_ Content Determination

H_2_O_2_ content was determined using the Solarbio Hydrogen peroxide (H_2_O_2_) content detection kit (Beijing Solarbio Science & Technology Co., Ltd. Beijing, China). Frozen plant samples were ground to powder in liquid nitrogen, then transferred to a new tube. A total of 1 mL of reagent 1 was added, then mixed well, 4 °C 8000 g/min for 10 min. The supernatant was collected on ice, 100 µL of reagent 2 and 200 µL of reagent 3 were added, then 4000 g/min for 10 min. The supernatant was discarded, and 1 mL of reagent 4 was added to the sediment, then determined at 415 nm.

Standard tube: A total of 1 mL of 1 mM H_2_O_2_, 100 µL of reagent 2, and 200 µL of reagent 3 were mixed in a tube, then 4000 g/min for 10 min. The supernatant was discarded, and 1 mL of reagent 4 was added to the sediment, then determined at 415 nm.

Control tube: A total of 1 mL of reagent 1, 100 µL of reagent 2, and 200 µL of reagent 3 were mixed in a tube, then 4000 g/min for 10 min. The supernatant was discarded, and 1 mL of reagent 4 was added to the sediment, then determined at 415 nm.

H_2_O_2_ content (μmol/g FW) = ∆A_sample tube_ ÷ ∆A_standard tube_ ÷ W.  ∆A_sample tube_ = A_sample tube_ − A_control tube_
 ∆A_standard tube_ = A_standard tube_ − A_control tube_

#### 4.2.8. SOD Activity Determination

SOD activity was determined using the Solarbio SOD activity detection kit (Beijing Solarbio Science & Technology Co., Ltd. Beijing, China). A 0.1 g sample was taken, 1 mL of extraction solution was added, mixed well, then 4 °C 8000 g/min for 10 min, then the supernatant was collected on ice. Then, the sample tube, control tube, blank tube 1, and blank tube 2 were prepared by adding reagents as described below, then 37 °C water bath for 30 min, then determined at 560 nm.

Sample tube: In total, 90 µL of supernatant prepared as before, 240 µL of reagent 1, 60 µL of reagent 2, 180 µL of reagent 3, 400 µL of H_2_O, 30 µL of reagent 4 were added to a tube and mixed well.

Control tube: A total of 90 µL of supernatant prepared as before, 240 µL of reagent 1, 180 µL of reagent 3, 400 µL of H_2_O, and 30 µL of reagent 4 were added to a tube and mixed well.

Blank tube 1: A total of 240 µL of reagent 1, 60 µL of reagent 2, 180 µL of reagent 3, 400 µL of H_2_O, and 30 µL of reagent 4 were added to a tube and mixed well.

Blank tube 2: In total, 240 µL of reagent 1, 180 µL of reagent 3, 400 µL of H_2_O, and 30 µL of reagent 4 were added to a tube and mixed well.

Inhibition percentage = [(A_blank 1_ − A_blank 2_) − (A_Sample tube_ − A_Control tube)_]/(A_blank 1_ − A_blank 2_) ×100%  SOD activity (U/g FW) = 11.11 × inhibition percentage/(1 − Inhibition percentage)/W × dilution ratio

#### 4.2.9. CAT Activity Determination

CAT activity was determined using the Solarbio CAT activity detection kit (Beijing Solarbio Science & Technology Co., Ltd. Beijing, China). A 0.1 g sample was taken, 1 mL of extraction solution was added, mixed well, then 4 °C 8000 g/min for 10 min, and the supernatant was collected on ice. Then a quartz cell was prepared, 1 mL of CAT testing working fluid and 35 µL of supernatant were added, mixed for 5 s, then determined at 240 nm immediately (A_1_) and at 240 nm after 60 s (A_2_).

CAT activity (U/g FW) = 678 × (A_1_ − A_2_) ÷ W

#### 4.2.10. Expression Analysis Using qRT-PCR

Frozen samples were ground to powder in liquid nitrogen. Total RNA was isolated using TriQuick Reagent (Beijing Solarbio Science & Technology Co., Ltd. Beijing, China), according to the manufacturer’s instructions. cDNA was synthesized using the HiFiScript cDNA Synthesis Kit (Jiangsu Cowin Biotech Co., Ltd., Taizhou, China). SuperStar Universal SYBR Master Mix (Jiangsu Cowin Biotech Co., Ltd., Taizhou, China) was used for qRT-PCR analysis. The gene-specific primers were selected from H.P. Chang et al. [39,42]. *TaRP15* was selected as the house-keeping gene for qRT-PCR analysis [44,45]. The sequences of primers used for qRT-PCR are listed in Supplementary Table S1.

#### 4.2.11. Statistical Analysis

Physiological, biochemical indexes, and phenotypic determination section: all significant differences among different wheat cultivars under control and salt treatment conditions were determined by one-way analysis of variance (ANOVA), and Tukey’s multiple range test was further performed for pairwise comparisons at a significance level of *p* < 0.05.

qRT-PCR section: significant differences between *Jimai 22* and *Liangxing 99* wheat cultivars were determined by the Student’s *t*-test.

## 5. Conclusions

In this study, we chose five wheat cultivars (*Ji 6358*, *Jimai 22*, *Keyi 5214*, *Liangxing 99*, and *Nongda 3432*) for research. Exposing the seedlings of the five wheat cultivars to NaCl concentrations from 0 to 150 mM revealed that *Liangxing* 99 and *Jimai* 22 exhibited superior salt tolerance. Under 100 mM and 150 mM NaCl treatments, *Liangxing* 99 and *Jimai* 22 showed higher chlorophyll, soluble sugar, and proline contents, higher SOD and CAT activities, and lower H_2_O_2_ and MDA levels. These results demonstrated that *Liangxing 99* and *Jimai 22* had higher osmotic adjustment capacity and lower oxidative injury under salt conditions. We then chose *Liangxing 99* and *Jimai 22* for further research. After 150 mM NaCl treatment, the transcript levels of abiotic stress-responsive gene *ZFP34*, antioxidant-related genes (*APX1*, *FSD2*, *APX2*), and photosynthetic gene *RbcS* in *Liangxing 99* and *Jimai 22* were examined. The results showed that after 150 mM NaCl treatment for 8 h, the expression levels of these genes were significantly higher in *Liangxing 99* than in *Jimai 22*. These results demonstrated that *Liangxing 99* displayed relatively strong salt tolerance among the five wheat cultivars evaluated in this study. While this study cannot substitute for field yield assessments under saline soils across the Huang–Huai–Hai region, it presents the first mechanistic characterization of seedling salt tolerance in five locally cultivated wheat cultivars. Among them, *Liangxing* 99 exhibited outstanding performance with strong osmotic adjustment ability, robust antioxidant defense, and pronounced upregulation of stress-responsive genes. This study supports the early screening of excellent salt-tolerant wheat cultivars at the seedling stage across the Huang–Huai–Hai region and delivers useful implications for developing wheat cultivars with stable productivity in saline farmlands.

## Data Availability

The original contributions presented in this study are included in the article/Supplementary Materials. Further inquiries can be directed to the corresponding author.

## Supplementary Materials

The following supporting information can be downloaded at: https://www.mdpi.com/article/10.3390/plants15121873/s1, Table S1: The sequences of primers used for qRT-PCR analysis.

## Abbreviations

The following abbreviations are used in this manuscript:

ROS	reactive oxygen species
MDA	malondialdehyde
SOD	superoxide dismutase
CAT	catalase
